# Dietary Components and Metabolic Dysfunction: Translating Preclinical Studies into Clinical Practice

**DOI:** 10.3390/nu8100632

**Published:** 2016-10-13

**Authors:** Gaetano Santulli

**Affiliations:** Herbert and Florence Irving Medical Center, Columbia University, New York, NY 10032, USA; gsantulli001@gmail.com

The importance of diet in the pathophysiology of metabolic syndrome is well acknowledged [1,2,3] and may be crucial in the determination of cardiovascular risk and the development of cardiovascular complications [4,5,6,7]. The contributions presented here provide an updated systematic overview examining in detail the functional role of different diets and dietary components in maintaining glucose homeostasis and prevention of long-term complications. The issue entitled “Diet and Metabolic Dysfunction” encompasses 40 peer-reviewed articles, both in the basic research field and in the clinical scenario, written by worldwide renowned experts. Intriguingly, one of the assets of the present issue is in the melting pot of researchers involved in this project, literally working in all continents, with contributions from United States, Canada, Mexico, Argentina, Italy, Ireland, Spain, Sweden, Austria, Liechtenstein, Germany, Japan, Korea, China, Hong Kong, Taiwan, Malaysia, Saudi Arabia, South-Africa, Nigeria, and Australia.

This Special Issue of Nutrients includes both evidence-based original research and state-of-the-art reviews and meta-analyses of the scientific literature. There are articles investigating different dietary regimens [8,9,10,11,12,13,14,15] and articles focusing on specific nutrients. In particular, we present studies on: omega-3 fatty acids [16], barley [17], honey [18], capsaicin [19], magnesium [20], selenium [21], fructose [22,23], vanillic acid [24], glutamine [25], histidine [26], isoleucine and valine [27], quercetin [28], rutin [29], naringin [30], red ginseng [31], epigallocatechin gallate (a component of green tea) [32], cudrania tricuspidata fruits [33], aloe vera [34], and probiotics and prebiotics [35]. Furthermore, given the increasing interest towards gut microbiota and metabolic syndrome [2,36,37], I decided to also accept in this Special Issue three interesting papers exploring this topic [38,39,40].

This collection of papers shows that the selection of foods should be based on scientific evidence, knowing the properties of each dietary component.
